# F-BAR family proteins, emerging regulators for cell membrane dynamic changes—from structure to human diseases

**DOI:** 10.1186/s13045-015-0144-2

**Published:** 2015-05-09

**Authors:** Suxuan Liu, Xinyu Xiong, Xianxian Zhao, Xiaofeng Yang, Hong Wang

**Affiliations:** Department of Cardiology, Changhai Hospital, Second Military Medical University, Shanghai, 200433 China; Center for Metabolic Disease Research, Department of Pharmacology, Temple University School of Medicine, Philadelphia, PA 19140 USA; Center for Cardiovascular Research, Department of Pharmacology, Temple University School of Medicine, Philadelphia, PA 19140 USA; Center for Thrombosis Research, Department of Pharmacology, Temple University School of Medicine, Philadelphia, PA 19140 USA

**Keywords:** F-BAR proteins, Membrane dynamics, Cellular functions, Pathophysiology

## Abstract

Eukaryotic cell membrane dynamics change in curvature during physiological and pathological processes. In the past ten years, a novel protein family, Fes/CIP4 homology-Bin/Amphiphysin/Rvs (F-BAR) domain proteins, has been identified to be the most important coordinators in membrane curvature regulation. The F-BAR domain family is a member of the Bin/Amphiphysin/Rvs (BAR) domain superfamily that is associated with dynamic changes in cell membrane. However, the molecular basis in membrane structure regulation and the biological functions of F-BAR protein are unclear. The pathophysiological role of F-BAR protein is unknown. This review summarizes the current understanding of structure and function in the BAR domain superfamily, classifies F-BAR family proteins into nine subfamilies based on domain structure, and characterizes F-BAR protein structure, domain interaction, and functional relevance. In general, F-BAR protein binds to cell membrane via F-BAR domain association with membrane phospholipids and initiates membrane curvature and scission via Src homology-3 (SH3) domain interaction with its partner proteins. This process causes membrane dynamic changes and leads to seven important cellular biological functions, which include endocytosis, phagocytosis, filopodium, lamellipodium, cytokinesis, adhesion, and podosome formation, via distinct signaling pathways determined by specific domain-binding partners. These cellular functions play important roles in many physiological and pathophysiological processes. We further summarize F-BAR protein expression and mutation changes observed in various diseases and developmental disorders. Considering the structure feature and functional implication of F-BAR proteins, we anticipate that F-BAR proteins modulate physiological and pathophysiological processes via transferring extracellular materials, regulating cell trafficking and mobility, presenting antigens, mediating extracellular matrix degradation, and transmitting signaling for cell proliferation.

## Introduction

Cell membrane curvature is a micro morphological change involved in many important cellular processes including endocytosis, phagocytosis, exocytosis, angiogenisis, and migration. The ability of cell membrane to achieve these dynamics is heavily determined by the collaboration between actin cytoskeleton and membrane-interacting proteins. Recently, a group of proteins named Fes/CIP4 homology-Bin/Amphiphysin/Rvs (F-BAR) domain family has emerged as the critical coordinators that regulate membrane curvature [1]. F-BAR proteins are membrane-associated proteins and regulate membrane curvature via binding to cell membrane phospholipids. F-BAR domain family is a member of the Bin/Amphiphysin/Rvs (BAR) domain superfamily which also includes the N-terminal amphipathic helix BAR (N-BAR) domain family and inverse BAR (I-BAR) domain family.

Recently, research has started to reveal potential functions for F-BAR proteins. However, the structures and functional diversities of the mammalian F-BAR family have not been elucidated. The mechanisms underlying F-BAR proteins regulating cell membrane dynamic changes and the pathophysiological roles of F-BAR proteins in human diseases are not clear. We present here a comprehensive analysis of the current understanding for structure, signaling, biological function, and pathophysiological association of the mammalian F-BAR family, and its association with diseases and development disorders in human and experimental animal models.

## BAR protein superfamily

**Figure 1:**
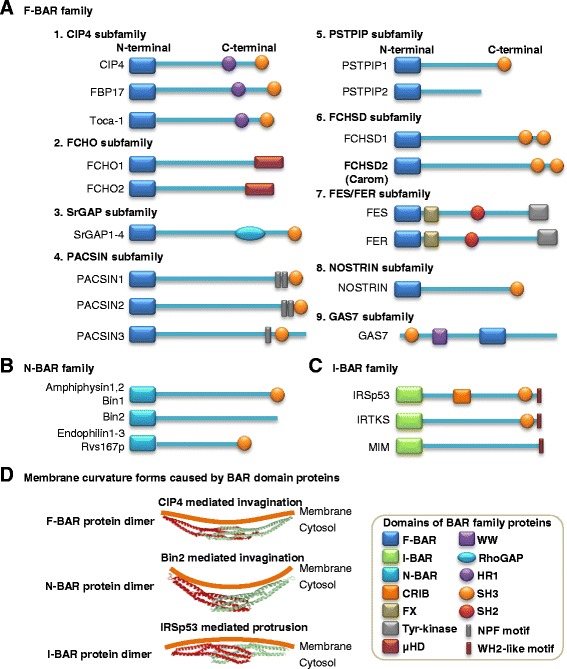
F-BAR, N-BAR and I-BAR family domain structure and membrane curvature models. BAR domain superfamily consists of three families based on distinct domain structures of F-BAR, N-BAR, and I-BAR. Bar domain proteins can form homodimer which binds to cell membrane leading to membrane curvature. **(A)** Domain structures of F-BAR family. F-BAR family has nine subfamilies determined by the specific domain combination. **(B)** Domain structures of N-BAR family. **(C)** Domain structures of I-BAR family. **(D)** Membrane curvature forms caused by BAR domain proteins. F-BAR, N-BAR, and I-BAR proteins bind to cell membrane and lead to different forms of curvature changes. Domain information is based on previous publications [1,3,4,7,8]. Crystal structure (a red monomer and a green monomer forming a dimer) is generated by using Protein Data Bank (PDB, http://www.rcsb.org/pdb/home/home.do). Abbreviations: BAR, Bin/Amphiphysin/Rvs; CRIB, CDC42-Rac interactive binding; F-BAR, Fes/CIP4 homology-BAR; FX, F-BAR extension; HR1, Protein kinase C-related kinase homology region 1; I-BAR, Inverse BAR; N-BAR, N-terminal amphipathic helix BAR; NPF, Asparagine proline phenylalanine; RhoGAP, Rho GTPase-activating protein; SH2, Src homology-2; SH3, Src homology-3; WH2, WASP homology 2; Tyr-kinase, Tyrosine kinase; μHD, μ-homology domain. Symbols listed in the framed box indicate representative domains.

The F-BAR protein was originally identified in a yeast two-hybrid system screen as CDC42-interacting protein 4 (CIP4) [2]. As indicated in Figure 1A, the N-terminal region of CIP4 was found to be highly conserved in several other proteins, such as tyrosine kinase FES and FES related (FER), thus termed as FES/CIP4 homology (FCH) domain. The FCH domain is next to a coiled-coil domain similar to BAR domain and constitutes a functional unit that together are termed as F-BAR domain. The F-BAR domain is evolutionarily conserved within eukaryotes and can bind to the negatively charged membrane phospholipids in lipid membranes to bridge the cytoskeleton and cell membrane.

The F-BAR family belongs to the BAR superfamily, which also includes N-BAR and I-BAR families and possesses a similar N-terminal BAR domain. At the C-terminal region, the BAR superfamily contains various combinations of different domains, such as Src homology-3 (SH3) domain, Src homology-2 (SH2) domain, tyrosine kinase domain, Rho GTPase-activating protein (RhoGAP) domain, WW domain, protein kinase C-related kinase homology region 1 (HR1) domain, and μ-homology domain (μHD) (Figure 1A–C). Particularly, the name “WW” domain refers to two strictly conserved tryptophans (W) in this domain. Based on literature information on domain characterization studies, we classified mammalian F-BAR family into nine subfamilies: CIP4, FCH only (FCHO), Slit-Robo GTPase-activating protein (srGAP), protein kinase C and casein kinase 2 substrates in neurons (PACSIN), proline-serine-threonine phosphatase-interacting protein (PSTPIP), FCH and double SH3 domain proteins (FCHSD), FES/FER, nitric oxide synthase traffic inducer (NOSTRIN), and growth arrest-specific 7 (GAS7) subfamilies [3-7].

The F-BAR, N-BAR, and I-BAR domain proteins can form a homodimer to generate a crescent-shaped structure with a family-specific radius of curvature [6] (Figure 1D). The distinctive feature of F-BAR domains is to bind to the membrane and form a shallow degree of invagination/concavity micro membrane morphologic change, with an arc depth ~3-fold smaller than those of N-BAR domains. The I-BAR domains are associated with a protrusion/convexity in the micro membrane morphologic change. Accordingly, F-BAR proteins and N-BAR proteins are normally involved in the invagination of membrane leading to endocytosis and phagocytosis [8]. I-BAR proteins are associated with the outwardly curved membranes for the formation of filopodium, lamellipodium, and angiogenesis [9].

Recently, F-BAR proteins have been identified as the novel and important coordinators that regulate not only endocytosis and phagocytosis but also filopodium, lamellipodium, cytokinesis, adhesion, and podosome formation. To fully understand the cellular functions of F-BAR protein family, it is important to analyze molecular basis, binding partners, and structure-function relationships of F-BAR proteins, and their functional implication.

## F-BAR proteins and membrane dynamics

As described above, F-BAR protein binds to phospholipids through the N-terminal F-BAR domain and bridges the membrane with cytoskeleton. However, we could not ignore the other domains of F-BAR protein that determine their specific function. We analyzed the structure of F-BAR proteins and proposed a modified characterization model for mammalian F-BAR family, which includes CIP4, FCHO, srGAP, PACSIN, PSTPIP, FCHSD, FES/FER, NOSTRIN, and GAS7 subfamilies.

**Figure 2:**
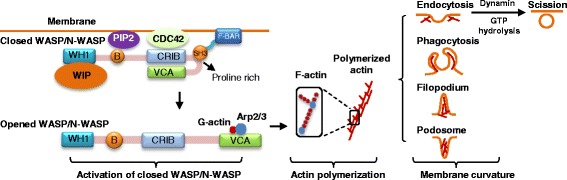
F-BAR protein binds to WASP/N-WASP via its SH3 domain to mediate membrane dynamic changes. F-BAR proteins possess a SH3 domain which binds to closed WASP/N-WASP and mediate various membrane dynamics. The SH3 domain of F-BAR protein binds to the proline-rich region of WASP/N-WASP that mediates the protein complex formation with PIP2, CDC42, and WIP, the B region of WASP binding to PIP2 while CRIB interacts with CDC42. The formation of protein complex results in the activation of closed WASP/N-WASP, leading to actin polymerization and the formation of various membrane curvature, including endocytosis, phagocytosis, filopodium, and podosome formation. Abbreviations: Arp2/3, Actin-related protein 2/3; B region, Basic region; N-WASP, Neural Wiskott-Aldrich syndrome protein; PIP2, Phosphatidylinositol (4,5)-bisphosphate; VCA, Verprolin, cofilin, acidic; WASP, Wiskott-aldrich syndrome protein; WH1, WASP homology 1; WIP, WASP interacting protein. For other abbreviations, refer to Figure 1. The diagram model is referenced from previous publications [5].

Most F-BAR proteins contain at least one C-terminal SH3 domain. The SH3 domain is an evolutionarily conserved protein-protein interaction domain of 50–60 amino acids. In general, F-BAR proteins bind to cell membrane via F-BAR domain association with membrane phospholipids (Figure 2). Through its SH3 domain, F-BAR proteins interact with proline-rich proteins like the Wiskott-Aldrich syndrome protein (WASP), neural (N)-WASP, and WASP family verproline-homologous protein (WAVE), which leads to WASP/N-WASP protein conformation change from a closed autoinhibitory conformation to an opened structure. The opened WASP/N-WASP protein exposes its C-terminal verprolin, cofilin, acidic (VCA) and CDC42-Rac interactive binding (CRIB) domains, which is activated and binds to actin-related protein 2/3 (Arp2/3) and G-actin, leading to the nucleation of filamentous actin (F-actin) and polymerization of actin (Figure 2) [10]. Actin polymerization causes various membrane curvatures, including endocytosis, phagocytosis, filopodium, and podosome. Some F-BAR proteins can further bind to the GTPase dynamin via SH3 domain, generate direct comet-like force by F-actin to push the membrane, and initiate the scission of vesicle [11].

## Structure and cellular functions of F-BAR family

**Figure 3:**
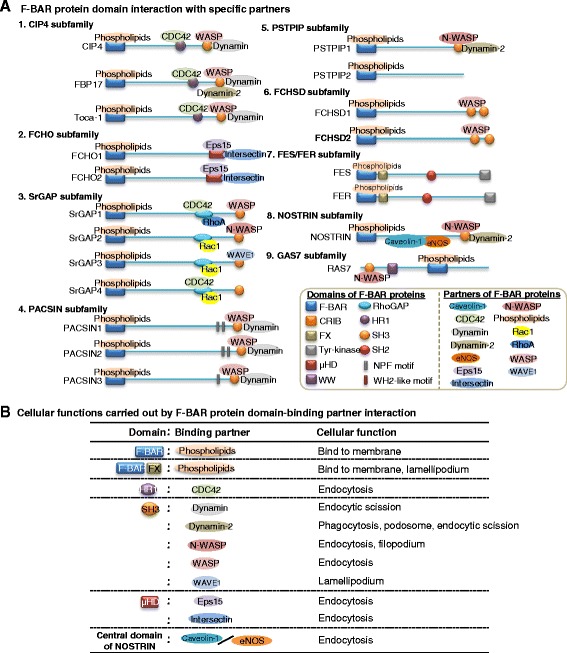
F-BAR protein domain interaction with specific partners and biological functions. Different domains of F-BAR proteins interact with specific binding partners **(A)** to perform various biological functions **(B)**, including binding to membrane, endocytosis, endocytic scission, lamellipodium, phagocytosis, filopodium, lamellipodium, and podosome. Abbreviations: eNOS, endothelial nitric oxide synthase; WAVE, WASP family-Verproline homologous protein. For other abbreviations, refer to Figures 1 and 2.

**Figure 4:**
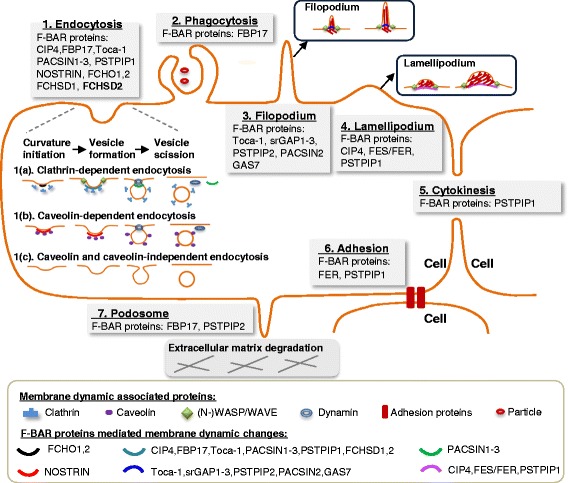
F-BAR protein-mediated membrane dynamic changes lead to seven major cellular functions. F-BAR proteins bind to cell membrane resulting in membrane curvature changes leading to seven major cellular functions. 1. Endocytosis consists of 1(a) clathrin-dependent endocytosis, 1(b) caveolin-dependent endocytosis, and 1(c) caveolin and caveolin-independent endocytosis. F-BAR proteins are found to be involved in clathrin or caveolin-dependent endocytosis. In clathrin-dependent endocytosis, FCHO1 and FCHO2 bind to cell membrane associated with clathrin to initiate curvature, while CIP4, FBP17, Toca-1, PACSIN1-3, PSTPIP1, and FCHSD1 and FCHSD2 mediate the formation of vesicle which involve other associated proteins including (N-)WASP or WAVE. PACSIN1-3 along with GTPase dynamin can bind to membrane and cause vesicle constriction, scission, and release. In caveolin-dependent endocytosis, NOSTRIN lead to the initiation and formation of caveolin-dependent endocytic vesicle. 2. Phagocytosis: FBP17 binds to the membrane to mediate the formation of phagocytosis in macrophage. 3. Filopodium: Toca-1, srGAP1-3, PSTPIP2, PACSIN2, and GAS7 can cause the formation of filopodium, which is a finger-like protrusion extended by the mobile edge of the cell. 4. Lamellipodium: CIP4, FES/FER, and PSTPIP1 lead to the formation of lamellipodium, which is a sheet-like protrusion on the mobile edge of the cell. 5. Cytokinesis: PSTPIP1 can migrate to the cleavage furrow to mediate cytokinesis, which is fundamental for the growth and development of all eukaryotic organisms. 6. Adhesion: FER and PSTPIP1 are involved in cell adhesion, which can mediate the process of immune response and the attachment of circulating inflammation cell to the blood vessel wall. 7. Podosome: FBP17 and PSTPIP2 mediate the formation of podosome, which regulates the extracellular matrix degradation. Symbols listed in the framed box indicate representative F-BAR proteins.

We further analyzed the domain structures, interactions, and specific binding partners of nine F-BAR protein subfamilies (Figure 3A), summarized the cellular functions carried out by different F-BAR protein domain-binding partner interactions (Figure 3B), described the procedure of F-BAR protein-mediated cellular function (Figure 4), and discussed the details below.

### CIP4 subfamily

CIP4 subfamily is a member of F-BAR domain proteins containing an F-BAR domain at the N-terminal, an HR1 domain in the middle, and a SH3 domain at the C-terminal. There are three CIP4-like proteins, CIP4, formin-binding protein 17 (FBP17), and transactivator of cytoskeletal assembly-1 (Toca-1).

CIP4 recruits WASP and GTPase dynamin via SH3 domain to participate in the initiation and scission of clathrin-dependent endocytic vesicle [12]. CIP4 has also been reported to be involved in endosomal trafficking. It is reported that the localization of CIP4 to endosomes was mediated in part via the curved phosphoinositide-binding face of the CIP4 F-BAR domain, that CIP4 is localized to early endosomes, and that its downregulation led to elevated epidermal growth factor receptor trafficking and cell cycle progress [13]. CIP4 is also found to inhibit neurite formation by producing lamellipodium in mouse cortical neurons, and it is dependent on the F-BAR and SH3 domain and its ability to multimerize [14]. Lamellipodium and Filopodium are dynamic actin-based membrane structures. Filopodium is necessary for neurite formation while lamellipodium may inhibit neurite formation [15,16].

FBP17, similar to CIP4, mediates the endocytosis of clathrin-dependent vesicle by recruiting WASP and dynamin for the vesicle initiation and scission [8]. FBP17 is associated with phagocytosis and podosomes in macrophages via recruiting WASP and dynamin-2 to membrane; the latter one is the ubiquitously expressed dynamin isoform [17]. The recruitment is the common molecular step required for the formation of podosomes and phagocytic cups.

Toca-1 has been shown to induce both endocytic vesicle and filopodium depending on CDC42/N-WASP-mediated actin polymerization [18]. Toca-1 is involved in endocytosis and filopodium and facilitates the coordination of membrane trafficking and morphology pathways. The induction of filopodium and neurite formation by Toca-1 can be inhibited by blockers of endocytosis. However, the potential mechanism of Toca-1 involved in filopodial formation and endocytic vesicle remains unclear.

### FCHO subfamily

FCHO include two members, FCHO1 and FCHO2, containing an N-terminal F-BAR and C-terminal μHD domain. FCHOs bind to the membrane via F-BAR domain and recruit the binding partners, Eps15 and intersectin, via its μHD domain to initiate a clathrin-dependent vesicle. FCHOs accumulate at the membrane before clathrin assembly and dissociate from the vesicle before it departs from the membrane (Figure 4). Both FCHO1 and FCHO2 are ubiquitously expressed. The expression of FCHOs was related with vesicle number and increased synaptic vesicle marker recycling. RNA interference (RNAi)-induced FCHO1 and FCHO2 reduction blocks endocytosis at early step [19].

### SrGAP subfamily

srGAP subfamily includes four members, srGAP1, srGAP2, srGAP3, and srGAP4, and are potentially involved in neuronal migration and angiogenesis. srGAP contains an N-terminal F-BAR domain, a central RhoGAP domain, and a C-terminal SH3 domain. SH3 domain binds to WASP in srGAP1, N-WASP in srGAP2, and WAVE1 in srGAP3 [20,21]. srGAP1–3 induce filopodium formation in mouse Neuro2a cells, similar to I-BAR protein IRSp53-induced membrane protrusion [22]. Therefore, srGAP subfamily is considered as “inverse F-BAR” (IF-BAR) to mediate membrane protrusion. Recently, srGAP4 has been reported to inhibit the outgrowth of hippocampal axons, and its F-BAR domain appeared to be more important for spatially localizing srGAP4 to axon growth cones [23]. It is suggested that hat srGAP1 binds to CDC42 and RhoA, that srGAP2 and srGAP3 interact with Rac1, and that srGAP4 binds to both CDC42 and Rac1 via RhoGAP domain to promote GTP hydrolysis [21,20,23].

### PACSIN subfamily

PACSIN, also known as syndapins, have three isoforms including PACSIN1, PACSIN2, and PACSIN3, all containing an N-terminal F-BAR domain, a C-terminal SH3 domain, and NPF motif. PACSIN are associated with WASP or dynamin via their SH3 domain, leading to WASP-dependent activation, dynamin-dependent scission, and eventually endocytosis [24]. The F-BAR domains of PACSIN1 and PACSIN2 also have a hydrophobic insertion loop, as found to promote endocytic vesicle scission. Overexpression of F-BAR domain of PACSIN2 caused microspike membrane changes in HeLa cells [25]. PACSIN2 is considered to facilitate the process of both endocytosis and filopodium-like formation.

### PSTPIP subfamily

PSTPIP subfamily consists of two members, PSTPIP1 and PSTPIP2. PSTPIP1 has an N-terminal F-BAR domain, PEST motifs (peptide sequence rich in proline, glutamic acid, serine, and threonine), and a C-terminal SH3 domain, whereas PSTPIP2 lacks the PEST motifs and SH3 domain. PSTPIP1 was initially cloned as CD2-binding protein 1 [26]. Similar to many other F-BAR proteins, PSTPIP1 binds to N-WASP and dynamin-2 to regulate endocytosis [27]. PSTPIP1 may have roles in CD2-induced T-cell adhesion and receptor-mediated signaling [28] and lamellipodium and cytokinesis regulation in COS cells [29]. PSTPIP2 is mostly expressed in macrophages and may regulate filopodium formation and motility in macrophages [30]. Knockdown of PSTPIP2 in macrophages promoted the assembly of FBP17 and subsequent formation of podosome, suggesting an antagonism between FBP17 and PSTPIP2 to regulate actin polymerization during podosome formation [31].

### FCHSD subfamily

FCHSD subfamily has two members, FCHSD1 and FCHSD2, each containing an F-BAR domain and two SH3 domains. FCHSD1 and FCHSD2 are mammalian orthologs of drosophila nervous wreck (Nwk)2 and Nwk1, which interact with WASP via its first SH3 and cooperate with CDC42 to regulate endocytic actin assembly at drosophila larval neuromuscular junction [32]. Nwk1 and Nwk2 present with similar activity of I-BAR proteins, generating protrusion of cellular membrane [33]. The F-BAR domains of FCHSD1 and FCHSD2 were found abundantly in the area of protrusion structures of human HEK293T cells, supporting their roles in facilitating membrane protrusion.

FCHSD2 gene encodes a protein termed as Carom. Carom binds to membrane-associated guanylate kinase inverted 1 (MAGI-1) or calcium/calmodulin-dependent serine protein kinase (CASK), in a competitive manner, via its distinct sequences of the C-terminal region [34]. CASK is reported to inhibit cell cycle progress, while MAGI-1 is indicated to inhibit the migration of cancer cell and is required for junctional cell adhesion [35,36]. It is possible that Carom may regulate cell growth, migration, and adhesion via complex formation with CASK or MAGI.

### FES/FER subfamily

The FES/FER subfamily includes two members, FES and FER, containing an N-terminal F-BAR domain, a central SH2 domain, and a C-terminal tyrosine kinase domain. The region adjacent to the F-BAR domain also binds to phospholipids, thus named the F-BAR extension (FX) domain.

The F-BAR and FX domains of FES/FER function as a membrane-binding module to bind to phospholipids and induce the membrane curvature for lamellipodium formation and cell motility [37]. FER also regulates the phosphorylation of F-actin-binding protein cortactin, leading to the efficient fibroblast migration and integrin-mediated cell adhesion [38].

### NOSTRIN

NOSTRIN contains an N-terminal F-BAR domain and a C-terminal SH3 domain. NOSTRIN binds to N-WASP through its C-terminal SH3 domain to facilitate the endocytosis and recruit dynamin-2 for vesicle scission [39].

NOSTRIN is highly expressed in endothelial cells and highly vascularized organs, binding to the oxygenase domain of endothelial nitric oxide synthase (eNOS) [40]. NOSTRIN could directly interact with caveolin-1 and eNOS to form a ternary complex in Chinese hamster ovary cells stably expressing eNOS [41]. NOSTRIN overexpression triggers the caveolin-dependent endocytosis to translocate eNOS away from the membrane and decrease eNOS activity.

### GAS7

GAS7 possesses an SH3 domain and a WW domain in the N-terminal, with its F-BAR domain positioned in the center region. Human GAS7 binds to N-WASP via SH3 domain to induce cell filopodium and regulate neurite outgrowth in differentiated brain cells [42]. Mouse GAS7 possesses a domain structure similar to human GAS7, but lacks the SH3 domain. Its WW domain can structurally resemble the human SH3 domain. Via WW domain, mouse GAS7 could interact with N-WASP and regulate the neurite outgrowth in hippocampal neurons [43].

## F-BAR protein-related functions in physiological and pathophysiological conditions

As discussed above, F-BAR proteins may modulate seven cellular functions: endocytosis, phagocytosis, filopodium, lamellipodium, cytokinesis, adhesion, and podosome formation (Figure 4 and Table 1). These seven cellular functions are critical cellular processes regulated in physiological and pathophysiological conditions.

**Table 1:** **Biological functions of F-BAR subfamilies**

**Proteins**	**Cellular functions**						
	**Endocytosis**	**Phagocytosis**	**Filopodium**	**Lamellipodium**	**Cytokinesis**	**Adhesion**	**Podosome**
1. CIP4 subfamily							
CIP4	√	N/A	N/A	√	N/A	N/A	N/A
FBP17	√	√	N/A	N/A	N/A	N/A	√
Toca-1	√	N/A	√	N/A	N/A	N/A	N/A
2. FCHOs subfamily							
FCHO1	√	N/A	N/A	N/A	N/A	N/A	N/A
FCHO2	√	N/A	N/A	N/A	N/A	N/A	N/A
3. srGAPs subfamily							
srGAP1	N/A	N/A	√	N/A	N/A	N/A	N/A
srGAP2	N/A	N/A	√	N/A	N/A	N/A	N/A
srGAP3	N/A	N/A	√	N/A	N/A	N/A	N/A
4. PACSINs subfamily							
PACSIN1	√	N/A	N/A	N/A	N/A	N/A	N/A
PACSIN2	√	N/A	√	N/A	N/A	N/A	N/A
PACSIN3	√	N/A	N/A	N/A	N/A	N/A	N/A
5. PSTPIPs subfamily							
PSTPIP1	√	N/A	N/A	√	√	√	N/A
PSTPIP2	N/A	N/A	√	N/A	N/A	N/A	√
6. FCHSDs subfamily							
FCHSD1	√	N/A	N/A	N/A	N/A	N/A	N/A
FCHSD2	√	N/A	N/A	N/A	N/A	N/A	N/A
7. FES/FER subfamily							
FES	N/A	N/A	N/A	√	N/A	N/A	N/A
FER	N/A	N/A	N/A	√	N/A	√	N/A
8. NOSTRIN subfamily	√	N/A	N/A	N/A	N/A	N/A	N/A
9. GAS7 subfamily	N/A	N/A	√	N/A	N/A	N/A	N/A

F-BAR proteins are implicated in various biological functions via interaction with specific binding partners. For F-BAR protein abbreviations, refer to Figure 1.

**Figure 5:**
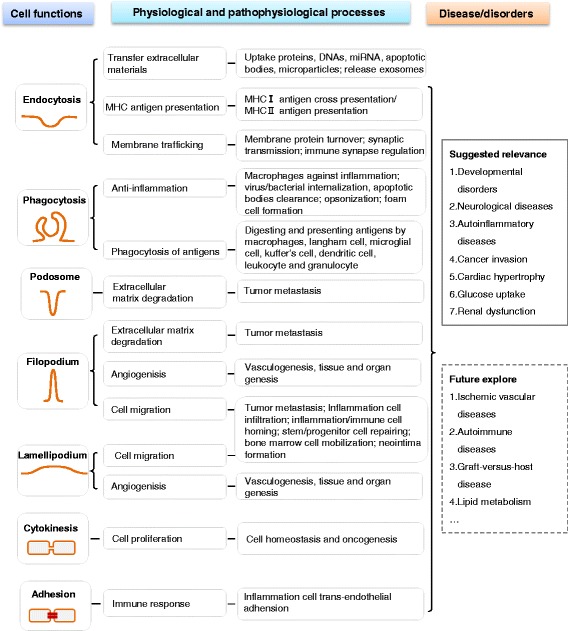
F-BAR protein-related functions in physiological and pathophysiological conditions. The cellular functions of F-BAR proteins include endocytosis, phagocytosis, filopodium, lamellipodium, cytokinesis, adhesion, and podosome formation. These cellular functions play important roles in many physiological and pathophysiological processes, including extracellular materials uptake, antigen presentation, exosome secretion, cell migration, angiogenisis, and so on. Until now, F-BAR proteins have been found to be related with many diseases and disorders, including 1. developmental disorders, 2. neurological diseases, 3. autoinflammatory diseases, 4. cancer, 5. cardiac hypertrophy, 6. glucose uptake, and 7. renal dysfunction. Accordingly, F-BAR proteins might also be associated with cardiovascular diseases, autoimmune diseases, graft-versus-host disease, and lipid metabolism, which need to be explored in the future.

*Endocytosis* is the most common cellular function mediated by F-BAR proteins and their binding partners. In general, F-BAR protein binds to the membrane phospholipids via its F-BAR domain and recruits WASP/N-WASP and dynamin via SH3 domain to regulate the initiation and scission of endocytic vesicle (Figure 3) in clathrin or caveolin-dependent ways (Figure 4). Endocytosis could uptake and process extracellular materials, including proteins, DNAs, miRNA, apoptotic bodies, and microparticles [44,45]. Endocytic compartments can be released from cells to form exosomes, which can be uptaken by other cells and can transfer the above functional materials between cells [46]. In addition, endocytosis is also involved in the presentation of soluble antigens, soluble major histocompatibility complex-II (MHC-II)-restricted antigens, and MHC-I antigen cross presentation [47] (Figure 5). Furthermore, endocytosis promoted membrane trafficking and neuronal membrane protein turnover [48].

*Phagocytosis* is an action of macrophages (Figures 4 and 5). Opsonization is a special phagocytic process by which the solid particle is coated with opsonins to facilitate the attachment and internalization of the particle, like clearance of apoptotic bodies by a professional phagocytic cell [49]. Phagocytosis of lipase-aggregated low density lipoprotein could promote the formation of lipid-laden macrophages, known as foam cells [50]. Similar to macrophages, there are many other macrophage-like cells involved in phagocytosis of antigens, including Langham cell, microglial cell, Kupffer cell, dendritic cell, leukocyte, and granulocyte [51,52].

*Filopodium* is a finger-like protrusion and is necessary for neurite formation [16] (Figure 4). Whereas, *lamellipodium* is characterized by a dense network of short and branched actin bundles and may inhibit neurite formation [15]. Filopodium and lamellipodium are considered as the major controllers of migration of normal cells and as the mediator of metastatic cancer cell invasion [53] (Figure 5). Filopodium- and lamellipodium-mediated migrations promote inflammatory and immune-cell infiltration and homing [54], and is the first step in stem/progenitor cell repairing, bone marrow cell mobilization, and neointima formation [55-57]. During angiogenisis, filopodium and lamellipodium drive endothelial tip cell to form different membrane structures for sprouting angiogenisis [58]. Furthermore, filopodium and lamellipodium are capable of probing the environment to sense the presence of attractive guidance cues and lead the way to vasculogenesis and even genesis of tissues and organs [59].

*Podosomes* primarily regulate extracellular matrix (ECM) degradation and is associated with tumor metastasis. Tumor metastasis requires tumor cells to break through the basement membrane and invade through dense networks of interstitial ECM proteins. Filopodium and lamellipodium may also regulate ECM degradation as they connect the cytoskeleton to ECM via focal contact points [60]. It is reported that PSTPIP1 mutation caused the transition from podosome to filopodium and increased filopodium-mediated ECM degradation in macrophages [61].

*Cytokinesis* is a key process during cell proliferation which is a crucial process of development, tissue repair, and oncogenesis. In homeostasis status, normal cells accomplish a constant balance between cell growth and death to maintain proper tissue and organ size and patterning [62]. During oncogenesis, the progression of cellular changes ultimately led to the uncontrolled proliferation of tumor [63]. Thus, cytokinesis could drive cell proliferation in different status to maintain normal cell homeostasis and tumor cell oncogenesis.

*Cell adhesion* is the binding of a cell to a surface or substrate, such as blood vessel wall or ECM, which can mediate immune response in different tissues. It is reported that endothelial cell adhesion molecules mediate the attachment of circulating inflammation cells to the blood vessel wall and subsequently their extravasation into perivascular tissues [64].

In summary, F-BAR proteins are involved in controlling endocytosis, phagocytosis, filopodium, lamellipodium, cytokinesis, adhesion, and podosome formation, and are suggested to be relevant to various diseases and disorder conditions (Figure 5). Based on the above functional analysis, we suspect that F-BAR proteins might also be involved in ischemic vascular diseases, autoimmune diseases, graft-versus-host disease, and lipid metabolism, which needs to be explored in the future.

## Roles of F-BAR proteins in diseases and developmental disorders

As indicated in Table 2, F-BAR expression change and gene mutation is associated with seven kinds of disease and disorder conditions, including developmental disorders, neurological diseases, autoinflammatory diseases, cancer invasion, cardiac hypertrophy, glucose uptake, and renal dysfunction.

**Table 2:** **Altered expression/mutation of F-BAR proteins in diseases and developmental disorders**

**Diseases and disorders**	**Species**	**F-BAR protein changes**	**PMID ID#**
Developmental and vascular disorders			
Embryonic defects	Drosophila, zebrafish	CIP4↑, PACSIN3↓	23424199, 19997509
Dorsoventral defects	Zebrafish	FCHO1↓	22484487
Vascular defects	Zebrafish	NOSTRIN↓	22751148
Postnatal retinal angiogenisis	Mice	NOSTRIN↓	22751148
Neurological disorders			
Neurodevelopmental disorders	Mice	srGAP2↓, srGAP3↓	23505444, 22820399
3p syndrome	Human	srGAP3 deletion	19760623
Huntington’s disease	Human	CIP4↑, PACSIN1↓	12604778, 23852340
Epileptic seizures	Mice	PACSIN1↓	21926968
Autoinflammatory diseases			
Wiskott-Aldrich syndrome	Human	FBP17↓	19155218
PAPA syndrome	Human	PSTPIP1 mutation	21532836
Chronic multifocal osteomyelitis	Mice	PSTPIP2 mutation	16122996
Cancers			
Bladder tumor	Human	FBP17↑	21421245
Breast tumor	Human	CIP4↓	21525036
Leukemia	Human	FBP17↑, FES/FER↑, *FCHSD2*↑	11438682, 22201778, 22902056
Cardiac hypertrophy	Rat	CIP4↑	23915320
Glucose uptake elevation	Rat, mice	CIP4↓, PACSIN3↑	19509061, 17320047
Renal dysfunction	Human	CIP4↑	22745576

F-BAR protein expression and mutations are observed in various disease and developmental disorders in human, mice, rat, zebrafish, and drosophila in publications cited by PMID#. FCHSD2 was found differentially expressed in a cardiovascular disease system in our recent study. Abbreviations: 3p syndrome, 3p25-p26 deletion syndrome; PAPA syndrome, Pyogenic arthritis, pyoderma gangrenosum, and acne syndrome.

### Developmental and vascular disorders

#### Developmental disorders

CIP4, PACSIN3, and FCHO1 are associated with developmental disorders. CIP4 is reported to inhibit actin nucleation of embryonic morphogenesis in drosophila embryogenesis [65]. Overexpression of CIP4 antagonized actin nucleation associated with Diaphanous (Dia), which is an actin nucleator responsible for F-actin formation in drosophila membrane compartmentalization. PACSIN3 may be related with early notochord formation during embryonic development in zebrafish, as axial mesodermal cell failed to migrate and the midline convergence of notochord precursors was defective in PACSIN3-deleted embryos [66]. FCHO1 was found interacting with Bmp receptor Alk8 and positively regulating Bmp signal transmission in dorsoventral patterning of zebrafish embryos [67].

#### Vascular disorders

Interestingly, NOSTRIN is reported to be necessary for proper vascular development in zebrafish and postnatal retinal angiogenesis in mice [68]. Knockdown of NOSTRIN in zebrafish embryos caused reduction of filopodium number and length and altered tip cell morphology, leading to abnormal intersegmental vessel trajectory. In NOSTRIN knockout mice, postnatal retinal angiogenesis was impaired due to the impairment of NOSTRIN-mediated fibroblast growth factor 2 signal transduction in endothelial cells, resulting in the suppression of endothelial tip cell migration.

### Neurological disorders

#### Neurodevelopmental disorders

Altered expression and mutation of F-BAR family was associated with neurological disorders in human and mice. The expression of srGAP3 was found to be reduced in childhood-onset schizophrenia proband [69]. srGAP3 knockout mice showed various behavioral phenotypes and complex neuroanatomical changes, including impaired spontaneous alternation and social behavior, enlarged lateral ventricles and spines, and increased basal activity of GTPase Rac1 [70]. It is reported that srGAP2 could act through srGAP3-Rac1 signaling to attenuate neuronal differentiation and neurite outgrowth in mouse neuroblastoma cells [22]. Overexpression of srGAP2C, srGAP2 with a truncated F-BAR domain, could impaire its function, leading to the neoteny of dendritic spine maturation in mouse neurons [71].

#### 3p syndrome

Distal 3p25-p26 chromosome deletion syndrome (3p syndrome) is a rare contiguous gene disorder characterized by low birth weight, mental retardation, telecanthus, ptosis, and micrognathia. srGAP3 was disrupted and functionally inactivated by a translocation breakpoint in a patient with 3p syndrome [72]. Microarray analysis of 14 patients with 3p syndrome revealed that srGAP3 was the major determinant of mental retardation [73].

#### Huntington’s disease

Huntington’s disease (HD) is caused by expansion of a polyglutamine repeat within the N-terminal region of huntingtin and present with severe neurodegenerative disorders. CIP4 interacted with huntingtin via SH3 domain, and overexpression of CIP4 induced the death of striatal neurons during HD pathogenesis [74]. PACSIN1 could also interact with huntingtin to interfere PACSIN1 mediated-endocytic removal of glutamate NMDA receptor subunit 3A (GluN3A), leading to age inappropriate synapse destabilization during HD pathogenesis [75].

#### Epileptic seizures

PACSIN1 complex with dynamin-1 may act as pivotal membrane anchoring factor for dynamin-1 during regeneration of synaptic vesicles. Gene deficiency of PACSIN1 or dynamin-1 in mice led to the development of epileptic seizures correlating with excessive hippocampal network activity [76].

### Autoinflammatory diseases

#### Wiskott-Aldrich syndrome (WAS)

WAS is an X chromosome-linked immunodeficiency disorder, characterized by eczema, thrombocytopenia, immune deficiency, and bloody diarrhea. WAS patients lack expression of WASP which binds to F-BAR protein FBP17 and regulates membrane dynamic changes (Figure 3). WAS patients presented with defects in forming WASP-FBP17-dynamin complex and podosomes/phagocytic cups in macrophages [17]. In addition, CIP4 may be related to WAS as CIP4-null mice developed thrombocytopenia, characterized by fewer proplatelet-like extensions and more rigid membrane, a phenotype observed in WAS patients [77].

#### PAPA syndrome

PAPA syndrome (pyogenic arthritis, pyoderma gangrenosum, and acne) is an autosomal dominant arising from PSTPIP1 gene mutation in the SH3 domain of PSTPIP1 [78]. It impaired PSTPIP1-WASP binding and induced the transition from podosome to filopodium formation. Increased filopodium formation leads to ECM degradation and enhanced invasive properties in PAPA syndrome.

#### Chronic multifocal osteomyelitis

Chronic recurrent multifocal osteomyelitis (CRMO) is a human autoinflammatory disorder that primarily affects bone, skin, or gastrointestinal tract. A missense mutation (L98P) of PSTPIP2 in mice led to pathophysiological changes similar to CRMO, called chronic multifocal osteomyelitis in mice [79] with increased IL-1β secretion in neutrophils and the inflammasome-independent IL-1β-mediated autoinflammatory reactions [80].

### Cancers

#### Bladder tumor

FBP17 was found expressed in three bladder tumor cell lines and primary bladder tumor cells from patients [81]. FBP17 knockdown significantly decreased the podosome formation and inhibited the invasive capacity in tumor cells [81].

#### Breast tumor

CIP4 suppressed Src-induced invasion in MDA-MB-231 breast tumor cells [82]. CIP4 knockdown cells inhibited endocytosis of type I matrix metalloprotease, leading to increased ECM degradation and breast tumor cell invasion.

#### Leukemia

Rearrangement of the mixed lineage leukemia (MLL) gene at chromosome 11q23 is commonly detected in leukemia. FBP17 is a fusion partner of the MLL gene at 11q23 and may be related to MLL [83]. FES/FER also implicated oncogenic KIT/FLT3 growth and survival signaling in leukemia [84]. Activated alleles of FES are potent inducers of myeloid differentiation. Knockdown of FCHSD2 enhanced chemosensitivity, whereas its overexpression increased cellular chemoresistance in U937 cells [85]. FCHSD2 levels are recognized as a clinical predictor for chemotherapy response in leukemia patients.

### Cardiac hypertrophy

It is suggested that CIP4 regulated intracellular hypertrophic signal transduction to control the growth of myocytes in heart disease. Knockdown of CIP4 inhibited myocyte hypertrophy and the inhibition could be rescued by expression of a recombinant CIP4 [86].

### Glucose uptake

CIP4 co-localized with glucose transporter (GLUT) 4 in L6 GLUT4 myc-expressing myoblasts [87]. Knockdown of CIP4 increased glucose uptake by elevating cell surface GLUT4. Overexpression of PACSIN3 in adipocytes caused an inhibition of GLUT1 endocytosis and induced GLUT1 membrane localization, leading to the elevation of glucose uptake [88].

### Renal dysfunction

CIP4 is highly expressed in tubular epithelia of 5/6-nephrectomized rat models and TGF-β1 treated human kidney (HK)-2 cells [89]. Overexpression of CIP4 promoted renal epithelial-mesenchymal transition (EMT) and induced ECM deposition in TGF-β1-treated HK-2 cells.

Finally, we summarize the F-BAR protein expression changes and potential mechanisms in disease and pathophysiological conditions in Table 3. F-BAR protein expression change and mutations are associated with developmental disorders, neurological and autoinflammatory diseases, cancer invasion, cardiac hypertrophy, glucose uptake, and renal dysfunction. We suspect that F-BAR proteins contribute to pathophysiological conditions via cell membrane dynamic modulation and subsequent cell function changes as described in Figure 5. F-BAR family proteins may provide novel potential therapeutic targets for neurological and autoinflammatory diseases, cardiovascular disorder, cancer, and metabolic disorders.

**Table 3:** **F-BAR proteins changes and potential mechanisms in diseases and pathophysiological conditions**

**Proteins**	**Changes**	**Disease/condition**	**Species**	**Mechanisms**	**PMID ID#**
1. CIP4 subfamily					
CIP4	↑	Embryogenesis	Drosophila	Inhibits actin nucleation associated with Diaphanous	23424199
	↑	Huntington’s disease	Human	Induces striatal neuron death	12604778
	↓	Breast tumor	Human	Suppresses Src-induced tumor cell invasion	21525036
	↓	Cardiac hypertrophy	Rat	Inhibits myocyte hypertrophy	23915320
	↓	Glucose uptake	Rat	Induces glucose uptake via GLUT4 endocytosis	19509061
FBP17	↑	Leukemia	Human	A fusion partner of mixed lineage leukemia	11438682
	↓	Bladder tumor	Human	Inhibits bladder tumor cell invasion	21421245
	↓	Wiskott-Aldrich syndrome	Human	Suppresses podosomes/phagocytic cup formation	19155218
2. FCHO subfamily					
FCHO1	↓	Dorsoventral defects	Zebrafish	Suppresses Bmp signal transmission	22484487
3.srGAP subfamily					
srGAP2	↓	Neuronal development	Mice	Suppresses neuronal development through srGAP3	23505444
srGAP3	↓	Neuronal development	Mice	Induces basal activity of Rac1	22820399
	Deletion	3p syndrome	Human	Gene is deleted, mechanism not analyzed	19760623
4. PACSIN subfamily					
PACSIN1	↓	Epileptic seizures	Mice	Induces hippocampal network activity	21926968
	↓	Huntington’s disease	Human	Induces age inappropriate synapse destablization	23852340
PACSIN3	↓	Embryonic defects	Zebrafish	Suppresses early formation of notochord	19997509
	↑	Glucose uptake	Mice	Induces glucose uptake via GLUT1 trafficking	17320047
5. PSTPIP subfamily					
PSTPIP1	Mutation	PAPA syndrome	Human	Gene mutation alters WASP activity	21532836
PSTPIP2	Mutation	Chronic multifocal osteomyelitis	Mice	Gene mutation and IL-1β induces inflammation	16122996
6. FCHSD subfamily					
FCHSD2	↑	Leukemia	Human	Increases leukemia chemoresistance	22902056
7. FES/FER subfamily					
FES/FER	↑	Leukemia	Human	Induces growth and survival signaling in leukemia	22201778
8. NOSTRIN subfamily					
NOSTRIN	↓	Postnatal retinal angiogenisis	Mice	Suppresses endothelial tip cell migration	22751148

F-BAR protein expression is observed in various disease and pathophysiological conditions via suggested mechanisms in human, mice, rat, zebrafish, and drosophila. Relevant publications are cited by PMID#. For other abbreviations, refer to Table 2.

## Future perspective

F-BAR proteins can recruit different binding partners to regulate membrane dynamics and cellular functions. Considering the structure features and functional implications of F-BAR proteins described above, we anticipate that F-BAR proteins modulate physiological and pathophysiological processes via transferring extracellular materials, regulating cell trafficking and mobility, presenting antigens, mediating ECM degradation, and transmitting signaling for cell proliferation. The continued efforts to explore fundamental details of F-BAR family structure, partner, signaling, and regulation would provide important insights to our understanding and to the identification of their therapeutic potential.

## Abbreviations

Arp2/3: Actin-related protein 2/3
BAR: Bin/Amphiphysin/Rvs
CIP4: CDC42-interacting protein 4
CRIB: CDC42-Rac interactive binding
ECM: Extracellular matrix
F-actin: Filamentous actin
F-BAR: Fes/CIP4 homology-Bin/Amphiphysin/Rvs
FCHO: FCH only
FCHSD: FCH and double SH3 domain proteins
FER: FES related
FX: F-BAR extension
GAS7: Growth arrest-specific 7
HR1: Protein kinase C-related kinase homology region 1
I-BAR: Inverse BAR
N-BAR: N-terminal amphipathic helix BAR
NOSTRIN: Nitric oxide synthase traffic inducer
N-WASP: Neural Wiskott-Aldrich syndrome protein
PACSIN: Protein kinase C and casein kinase 2 substrates in neurons
RhoGAP: Rho GTPase-activating protein
SH2: Src homology-2
SH3: Src homology-3
srGAP: Slit-Robo GTPase-activating protein
μHD: μ-homology domain
VCA: Verprolin, cofilin, acidic
WASP: Wiskott-Aldrich syndrome protein
WAVE: WASP family verproline-homologous protein
